# Partial splenic embolization as management of portal hypertension-related hypersplenism in pediatric patients

**DOI:** 10.3389/fradi.2026.1731300

**Published:** 2026-03-18

**Authors:** Karen Juliana Salinas-Castro, Valentina Mejia-Quiñones, Santiago Quiceno-Ramirez, Juan Sebastian Toro-Gutierrez, Verónica Botero, Luis Alfonso Bustamante-Cristancho, Alfonso José Holguín-Holguín

**Affiliations:** 1 Facultad de Ciencias de la Salud, Universidad Icesi, Cali, Colombia; 2 Centro de Investigaciones Clínicas, Fundacion Valle del Lili, Cali, Colombia; 3 Departamento de Radiología e Imágenes Diagnósticas, Fundacion Valle del Lili, Cali, Colombia; 4 Departamento de Gastroenterología Pediátrica, Fundacion Valle del Lili, Cali, Colombia; 5 Departamento de Ciencias Clínico-Quirúrgicas, Pontificia Universidad Javeriana, Cali, Colombia

**Keywords:** pediatric, portal hypertension, spleen, therapeutic embolization, thrombocytopenia

## Abstract

**Introduction:**

Endovascular partial splenic embolization (PSE) represents a therapeutic alternative for managing portal hypertension and hypersplenism that preserves splenic parenchyma and maintains immune function. This study aimed to describe the therapeutic response and safety profile of PSE in pediatric patients treated over an eleven-year period at a tertiary care center in Colombia.

**Methods:**

This was a descriptive, retrospective case series. We included 12 pediatric patients aged 8–15 years with hypersplenism secondary to portal hypertension who underwent PSE between 2012 and 2023 at our institution. Hematological parameters, endoscopic findings, and post-procedural complications were analyzed.

**Results:**

The median age was 13 years; nine patients (75%) had portal hypertension and hypersplenism due to portal vein thrombosis, and three (25%) due to hepatic fibrosis. The median percentage of embolized splenic parenchyma was 40% [IQR (25–60)]. Baseline platelet counts were 58,000 × 10⁹/L (50,250–67,000), and baseline leukocyte counts were 3,485 × 10⁹/L (2,728–4,090). Following PSE, statistically significant increases (delta) were observed in platelet count (median 235,000 × 10⁹/L, 95% CI 119,500–450,500; *p* = 0.0004) and white blood cell count (median 5,600 × 10⁹/L, 95% CI 2,883–8,925; *p* = 0.002). Eight patients underwent endoscopic follow-up; six demonstrated improvement in esophageal varices grade. Complications included transient abdominal pain and fever. No major adverse events, such as splenic abscess, infarction, rupture or hematoma, were recorded.

**Conclusions:**

In our series, PSE demonstrated safety and efficacy in managing portal hypertension-related hypersplenism in pediatric patients, with significant improvements in hematological parameters and esophageal varices grade in most cases. PSE represents a valuable minimally invasive alternative to splenectomy, preserving splenic function while achieving therapeutic goals, even in patients requiring repeat interventions.

## Introduction

Increased portal venous pressure causes retrograde flow and blood congestion in the spleen, leading to splenomegaly. This results in sequestration and accelerated destruction of blood components, a phenomenon known as hypersplenism (1–3). Hypersplenism often results from portal hypertension, which is defined as an increase in venous pressure within the portal system, determined by a hepatic venous pressure gradient greater than 5 mmH (4). In the pediatric population, etiologies are diverse and include conditions such as cirrhosis, biliary atresia, and extrahepatic biliary obstruction (4). Preserving the spleen’s essential role in immunity, particularly against encapsulated bacteria, is crucial in managing this condition (3).

Interventional treatment for hypersplenism evolved from total splenic artery embolization to the spleen-preserving technique of partial splenic embolization (PSE), which was developed by Spigos et al. in 1979 to reduce morbidity and mortality (5). In the pediatric population, PSE has been proposed as an effective alternative to splenectomy for refractory hypersplenism associated with portal hypertension (2, 6–12), with complications including post-embolization syndrome, splenic abscesses, and infarction (13–15).

Despite the established efficacy of PSE in managing hypersplenism, controlling variceal bleeding, and promoting esophageal varices regression secondary to portal hypertension (16), its application and supporting evidence in our local pediatric population remain limited. Therefore, this study aims to evaluate the treatment response to PSE in pediatric patients with portal hypertension-related hypersplenism, specifically focusing on post-procedure hematological and endoscopic alterations.

## Methods

This retrospective case series included patients under 18 years of age with symptomatic hypersplenism (defined by persistent thrombocytopenia and/or leukopenia) secondary to portal hypertension who underwent PSE between 2012 and 2023. Cases were identified using the institutional interventional radiology database and ICD-10 codes associated with thrombocytopenia, with records filtered according to inclusion criteria and database management performed in REDCap. Patients with traumatic splenomegaly and those with incomplete medical records were excluded. Patients with portosystemic shunts were included if they exhibited refractory hypersplenism despite the shunt being patent or if the shunt was not deemed sufficient to control the splenomegaly or cytopenia.

Primary outcomes included the variance in platelet and leukocyte counts post-PSE compared to baseline. Secondary outcomes include the rate of major complications, and improvement in the grade of esophageal varices upon endoscopy.

Data collection involved review of electronic medical records, imaging studies (ultrasound and angiography), and pre- and post-procedural laboratory tests.

The study was approved by the institutional ethics committee (No. 2098), with complete data anonymization.

### Statistical analysis

Normality was assessed using the Shapiro–Wilk test. Continuous variables were expressed as mean ± standard deviation or median with interquartile range (IQR), depending on distribution, and categorical variables as frequencies and percentages. To compare platelet and leukocyte counts, the Wilcoxon signed-rank test was used. Comparisons were performed between baseline and immediate post-procedure for both platelet and leukocyte counts, and additionally between baseline and 3 months for platelet counts only. Median differences, 95% confidence intervals, and *p*-values were calculated. Statistical significance was set at *p* < 0.05. Statistical analysis was performed using RStudio software.

### Procedure

**Figure 1:**
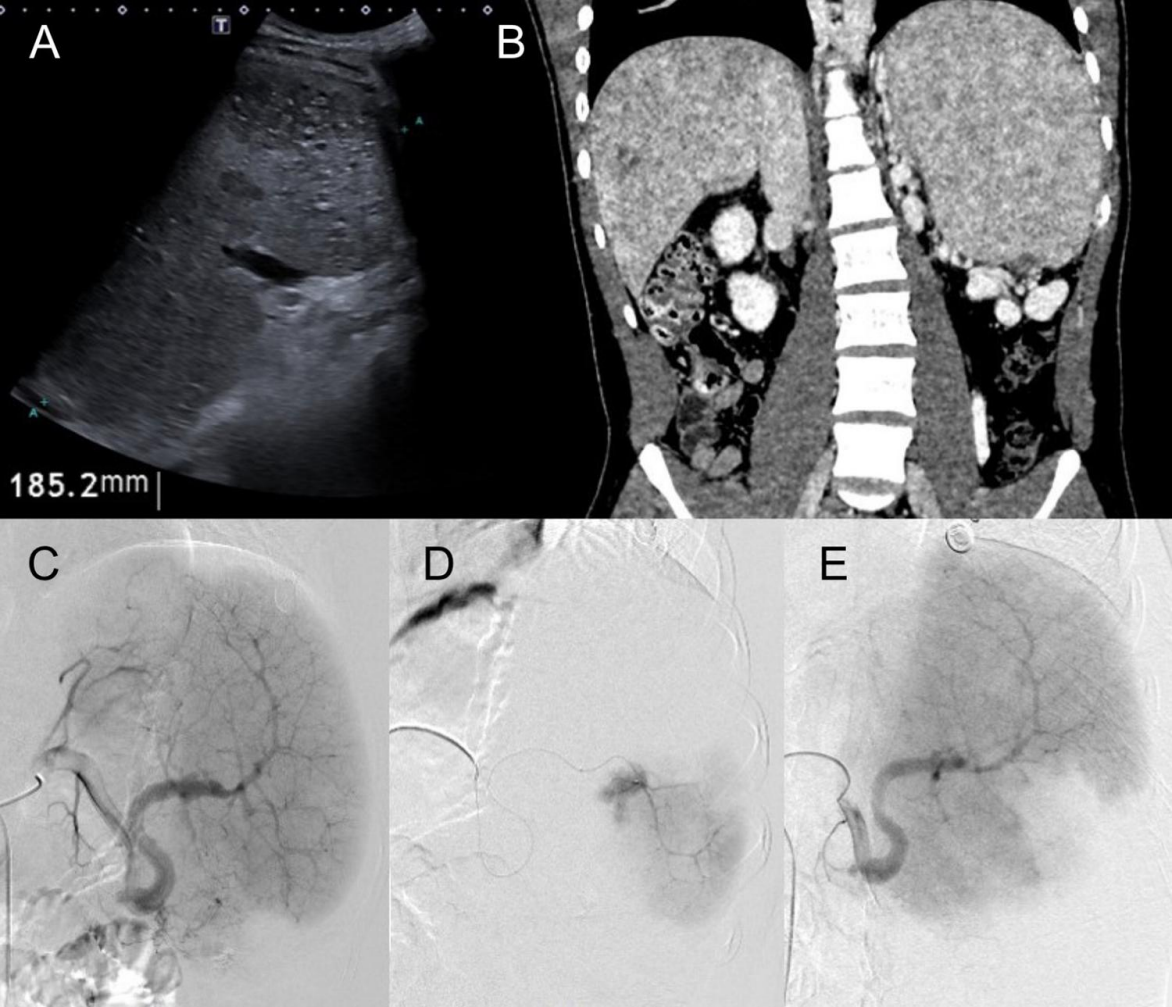
**(a)** Total abdominal ultrasound and **(b)** abdominal angiotomography in multiplanar reconstruction in coronal section in portal phase showing splenomegaly. **(c)** Angiography of the spleen from the splenic artery. **(d)** Superselective angiography in the middle third of the spleen showing healthy parenchyma. **(e)** Absence of contrast in the middle third of the spleen demonstrating its embolization.

All embolizations were performed under general anesthesia using a percutaneous transfemoral approach. Splenic artery catheterization was achieved using a 5-Fr cobra catheter and a 0.035-inch hydrophilic guidewire. Selective embolization was performed using a Progreat microcatheter with 350–500 μm polyvinyl alcohol (PVA) microparticles. The choice of PVA was predicated on its mechanism of action, aiming to achieve permanent occlusion within the most distal aspects of the splenic vascular bed. Furthermore, PVA was selected as a cost-effective embolic agent, making it a sustainable and effective option within the economic constraints of a developing nation. Pre- and post-embolization angiography was performed to confirm technical success and ensure the preservation of residual perfusion. In cases where the percentage of embolized parenchyma was not documented intraoperatively, it was estimated retrospectively by measuring the embolized splenic length and applying a proportional extrapolation based on quadrant division (Figure 1).

Periprocedural management included prophylactic antibiotic therapy with piperacillin–tazobactam and amikacin, adjusted according to body weight. Sedation and analgesia were achieved using anesthetic agents administered under continuous monitoring. The most frequently used regimen consisted of lidocaine (local infiltration), propofol (continuous intravenous infusion), and remifentanil (continuous intravenous infusion). Anesthetic selection, however, was tailored to patient characteristics and intraoperative requirements, and this combination was not applied uniformly in all cases.

## Results

### Clinical features

Partial splenic embolization (PSE) was performed in 12 pediatric patients with a median age of 13 years [IQR (8.8–15.3)]. Seven patients (58%) were female and five (42%) were male. All patients had portal hypertension; four (33%) presented with ascites, three (25%) had a history of variceal bleeding, and two (17%) had portosystemic shunts. The predominant etiology was portal vein thrombosis in nine patients (75%). No patients had splenic vein thrombosis or Budd–Chiari syndrome (Table 1).

**Table 1:** Baseline clinical and imaging characteristics (*N* = 12).

Variable	Value (*n*, %) or median (IQR)
Demographics	
Age, median (IQR)	13.0 (8.8–15.3)
Female sex	7 (58%)
Characteristics prior to embolization	
Portal hypertension	12 (100%)
Ascites	4 (33%)
Systemic port shunt	2 (17%)
Variceal bleeding history	3 (25%)
Previous partial splenic embolization	1 (8%)
Etiology	
Portal vein thrombosis	9 (75%)
Hepatic fibrosis	3 (25%)
Variceal features	
Esophageal varices	9 (75%)
With prior bleeding	3 (25%)
Prior band ligation	6 (50%)
Prior sclerotherapy	3 (25%)
Gastric varices	6 (50%)
With prior bleeding	0 (0%)
Pre-procedural Imaging Findings	
Signs of chronic liver disease	5 (42%)
Portal venous flow, cm/s	
Without thrombosis (*N* = 2)	35.8 (35.1–36.4)
With thrombosis (*N* = 4)^a^	22 (19.2–24)
Hepatic artery peak systolic velocity, cm/s (*N* = 6)	62.5 (43–76)
Spleen maximum diameter (US), cm (*N* = 8)	14.5 (12.1–16.2)

a Measured from patent intrahepatic or porto-systemic collateral vessels.

Nine patients (75%) had evidence of esophageal varices prior to embolization. Of these nine patients, three (33%) had a history of variceal bleeding (25% of the total cohort). Six patients had previously undergone variceal band ligation, and three had undergone sclerotherapy.

### Imaging characterization prior to embolization

Pre-procedural imaging revealed chronic liver disease changes in five patients. Six patients had previous ultrasound studies demonstrating increased mean portal vein velocity, consistent with portal hypertension (Table 2).

**Table 2:** Procedural characteristics.

Variable (*N* = 12)	Value (*n*, %) or median (IQR)
Upper pole embolization	1 (8.3%)
Middle portion embolization	3 (25%)
Lower pole embolization	11 (91.7%)
Estimated percentage of spleen embolization, median (IQR)	40 (28-52.8)
Use of polyvinyl alcohol (PVA)	12 (100%)
Periprocedural antibiotic (piperacillin–tazobactam, weight-adjusted)	12 (100%)

In the nine patients with portal vein thrombosis, the portal flow measurements (in the 6 reported cases) were obtained from patent intrahepatic or porto-systemic collateral vessels to assess dynamic flow characteristics in the presence of cavernous transformation.

### Procedure characteristics

Selective embolization was successfully achieved in all patients. The embolized splenic regions included the upper pole in one patient (8.3%), the middle portion in three patients (25%), and the lower pole in 11 patients (91.7%). Polyvinyl alcohol particles measuring 350–500 μm were used as the sole embolic agent in all patients, without adjunctive use of cyanoacrylate or coils (Table 2).

### Follow-up outcomes

All patients had pre-embolization laboratory records, with median values demonstrating leukopenia and thrombocytopenia. Following partial splenic embolization, statistically significant increases were observed in platelet counts (*p* = 0.0004) and leukocyte counts (*p* = 0.002). Specific median delta values, interquartile ranges, and 95% confidence intervals are presented in Table 3.

**Table 3:** Paraclinical and endoscopic follow-up indices.

Variable	Median (IQR)	95% CI	*p*-value
Baseline laboratory values			
Hemoglobin, g/dL	11.7 (10.9–12.7)	—	—
Hematocrit, %	35.1 (33.8–37.3)	—	—
Leukocytes, ×10⁹/L	3,485 (2,728–4,090)	—	—
Platelets, ×10⁹/L	58,000 (50,250–67,000)	—	—
Post-procedural changes			
*Δ* Platelets, immediate post vs. baseline	235,000 (91,500–345,500)	119,500–450,500	0.0004
Days to peak platelet count	14.5 (12.5–18.0)	—	—
*Δ* Leukocytes, immediate post vs. baseline	5,600 (3,615–7,562)	2,883–8,925	0.002
Days to peak leukocyte count	5.0 (1.0–15.5)	—	—
Endoscopic follow-up (*N* = 8)			
Median follow-up time (IQR)	232.5 (32.7–368.5)	—	—
Improvement in digestive varices	6/8 (75%)	—	—
Progression of digestive varices	2/8 (25%)	—	—

**Figure 2:**
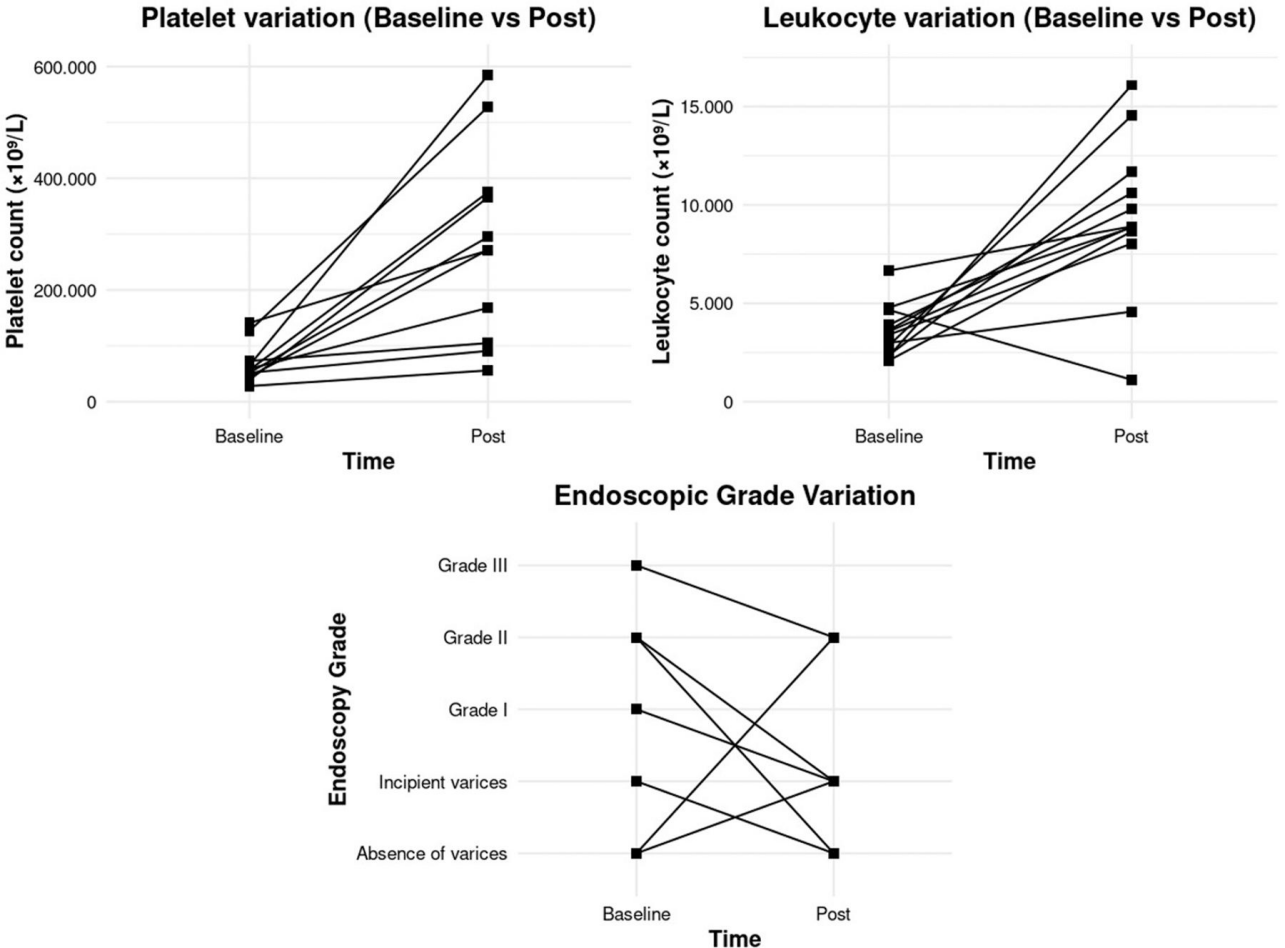
Paraclinical and endoscopic follow-up after partial splenic embolization. Individual trends in platelet count, white blood cell count, and degree of digestive varices are shown in the pre-procedure and follow-up periods (time points correspond to baseline and post-procedure follow-up).

Individual patient evolution is illustrated in Figure 2 using paired line graphs. Consistent increases in both platelet and leukocyte counts were observed after partial splenic embolization in most patients immediately post-procedure. Only eight patients underwent endoscopic follow-up, six (75%, median follow-up time = 232, 5 days) demonstrated improvement in esophageal varices grade following partial splenic embolization.

### Complications

Four patients (33%) presented with transient abdominal pain, which was difficult to control in three cases; however, all symptoms resolved with intravenous analgesia prior to discharge. Two patients (17%) developed fever, and one patient (8%) experienced post-embolization syndrome. The patient who underwent embolization of the splenic upper pole developed mild and asymptomatic pleural effusion following the procedure, which resolved spontaneously prior to discharge. No patients experienced major complications such as bleeding, splenic rupture, or superimposed infection. During outpatient follow-up, one patient (8%) experienced recurrent variceal bleeding five months after the procedure (Table 4).

**Table 4:** Post-procedure complications.

Complication (*N* = 12)	Value (*n*, %)
Abdominal pain	4 (33%)
Difficult-to-control abdominal pain	3 (25%)
Fever	2 (17%)
Post-embolization syndrome	1 (8%)
Left-sided pleural effusion	1 (8%)
Relapse due to gastrointestinal bleeding	1 (8%)
Major adverse events (infection, rupture, bleeding)	0 (0%)

## Discussion

Partial splenic embolization offers the advantage of preserving splenic parenchyma and immune function, avoiding total splenectomy, and has been proposed as an effective alternative in severe refractory thrombocytopenia. Vittorio et al. have highlighted its usefulness in managing hypersplenism associated with portal hypertension, emphasizing hematological improvement and reduction of hemorrhagic complications as key benefits (1). In the pediatric population, its use remains limited, but our eleven-year experience demonstrates sustained increases in platelet and leukocyte counts, consistent with international series, which supports its reproducibility (1, 6–11, 17).

The implementation of elective PSE at our institution evolved from our experience in the emergency management of splenic trauma. Historically, we observed a consistent and significant increase in platelet counts following post-traumatic splenic embolization. This clinical observation served as the physiological rationale to extrapolate the technique in a multidisciplinary collaboration with the pediatric gastroenterology department to the elective management of patients with portal hypertension and hypersplenism.

The technique used at our institution was consistent with that reported by other authors: transfemoral approach, selective catheterization of the celiac trunk and superselective catheterization of intraparenchymal branches, preferentially targeting the lower pole, with embolization using coils, gelatin sponge, autologous clots, or PVA particles of 350–500 μm (6–9, 12). The predominant use of these particles and size at our center reflected a clinical strategy aimed at achieving permanent and distal occlusion within the splenic vascular bed. This approach is particularly relevant to prevent recanalization via collateral circulation. Furthermore, the historical preference for PVA in our series aligns with the socioeconomic reality of a resource-limited setting. In this context, PVA represents a cost-effective and sustainable alternative to more expensive embolic devices, allowing for high technical success rates despite the economic constraints often encountered in a developing nation.

During endoscopic follow-up, significant reduction in esophageal varices grade was observed after partial splenic embolization. This regression may be considered an indirect marker of hemodynamic improvement in pediatric portal hypertension, and was associated with statistically significant and sustained increases in platelet and leukocyte counts, suggesting sustained hemodynamic and functional benefits. Beyond secondary prophylaxis, emergency PSE serves as a critical life-saving intervention for acute, catastrophic bleeding events in children. It has proven highly effective in controlling severe gastric variceal bleeding when standard medical management fails (18), and has been successfully utilized to achieve rapid hemodynamic stabilization in patients with life-threatening ectopic duodenal varices, thereby facilitating safe and effective combined therapy with endoscopic sclerotherapy (19). Similar findings have been reported in series where variceal regression decreases bleeding risk and reduces the need for subsequent endoscopic interventions (1, 6, 8).

A relevant finding was the successful repetition of PSE in a patient with a history of pulmonary hypertension, hepatopulmonary syndrome, and hypertensive gastropathy requiring esophageal variceal ligation. On this occasion, he consulted for epistaxis and episodes of hematochezia. Upon admission, he had platelet counts of 43,000 × 10⁹/L and leukocyte counts of 2,900 × 10⁹/L. Due to persistent gastrointestinal bleeding, it was decided to perform partial embolization of the spleen, which in subsequent paraclinical tests showed a platelet increase of up to 174,000 × 10⁹/L. This case further corroborates evidence that PSE serves as a highly effective and repeatable intervention for managing refractory esophageal variceal bleeding, particularly in pediatric cases of portal vein thrombosis where standard endoscopic band ligation has proven insufficient (20). This supports Vittorio's description of the feasibility of repeating the procedure in selected cases, optimizing cytopenia control without resorting to splenectomy (1).

In more than 90% of cases, the lower pole was embolized; the only embolization of the upper pole was associated with pleural effusion, consistent with the literature. Complications were limited to transient abdominal pain (33.3%), with no major events, which contrasts with reports describing fever, severe pain, and splenic abscesses (14–16, 18). This safety profile could be explained by the lower percentage of parenchyma treated, in line with the observation by Spigos et al. (5) that a smaller extent of embolization is associated with lower morbidity and mortality.

This study has several limitations that should be considered when interpreting the results. Firstly, its retrospective, single-center design introduces an inherent risk of selection bias and confounding factors that could not be fully controlled for, as well as limitations in complete data collection. Secondly, the small sample size and lack of a control group significantly limit the statistical power of the analysis and the generalizability of our findings to broader populations. Furthermore, the follow-up was heterogeneous and lacked long-term data, precluding any definitive assessment of the durability of the observed outcomes. Specific to the secondary outcomes, only eight patients had documented endoscopic follow-up, and the lack of direct portal pressure measurements necessitates caution when interpreting variceal regression as an indirect marker of hemodynamic improvement. Lastly, the estimation of embolized splenic parenchyma percentage was performed retrospectively through imaging, which may have introduced measurement bias.

## Conclusion

Our results reinforce the usefulness of PSE as a safe and promising strategy in the management of thrombocytopenia and leukopenia secondary to hypersplenism in the pediatric population. This procedure not only improves hematological parameters but also reduces the degree of digestive varices, which may translate into a lower risk of bleeding and a reduced need for endoscopic interventions. With a low rate of major complications and the possibility of repetition in selected cases, PSE represents a valid alternative to splenectomy in carefully selected patients.

## Data Availability

The raw data supporting the conclusions of this article will be made available by the authors, without undue reservation.
